# The crime, mental health, and economic impacts of prearrest diversion of people with mental health problems: A systematic review

**DOI:** 10.1002/cbm.2112

**Published:** 2019-04-10

**Authors:** Karen Schucan Bird, Ian Shemilt

**Affiliations:** ^1^ EPPI Centre UCL Institute of Education London UK

## Abstract

**Background:**

Prearrest diversion strategies are being adopted across the Western world, enabling the police to identify and divert people suspected of having mental disorder towards health and community services rather than the criminal justice system.

**Aims:**

To quantify longer‐term criminal justice and mental health outcomes after prearrest diversion of people with suspected mental disorder and consider economic correlates.

**Methods:**

A systematic review of published literature on longer term outcomes after prearrest diversion.

**Results:**

Only two quasi‐experimental studies, with four independent samples, could be included. Findings for criminal and mental health outcomes were inconclusive, but potential for adverse outcomes was identified. Ten studies with cost data suggested that prearrest diversion can lead to overall cost savings.

**Conclusions:**

There is still inadequate evidence on which to base prearrest diversion programmes. Although some benefits have been identified by the review, so have possible harms. Future research and funding strategies must build in high‐quality, systematic evaluation of outcomes before implementing a theoretically attractive strategy more widely.

## 1 INTRODUCTION

Responding to people who appear to have a mental disorder has been increasingly recognised as part of the *core business of policing* (Adebowale, 2013: 11), reflected in the high volume of interactions between the police and such people in the Western world. Studies in North America report between 1 and 20% of all police calls for service involve people with mental disorder (Livingston, 2016). Estimates in the United Kingdom suggest that up to 40% of police time involves a mental health element (Home Affairs Select Committee, 2015). People with mental health problems are more likely to become victims of crime compared with the general population (Pettitt et al., 2013) and more likely to be arrested for minor offences (Charette, Crocker, & Billette, 2014). Within police custody, small‐scale studies suggest that up to 39% of individuals have some kind of mental illness (McKinnon, Thomas, Noga, & Senior, 2016: 218). Some have even suggested that the intersections between police and people with mental disorder are indicative of the *criminalisation of mental illness* (Butler, 2014; Reuland, Schwarzfeld, & Draper, 2009; Teplin, 1985). The estimated economic implications amount to £1.6 billion annually spent on arresting, convicting, imprisoning, and criminal justice supervision of people with mental disorder (Corner, Jones, & Honeyman, 2007).

Following the Bradley Report (2009) about people with mental health problems or learning disabilities in the criminal justice system in England and Wales, policing and mental health attracted particular policy attention there (Home Affairs Select Committee, 2015), although these issues are far from unique to the United Kingdom. Improved prearrest/booking strategies were planned as part of the broader effort to improve police interactions with such people, enabling officers to identify and divert them to assessment and treatment services rather than arrest them. Appropriateness of diversion is likely to be based on the seriousness of the offence, the safety of the individual and the public (DeMatteo, LaDuke, Locklair, & Heilbrun, 2013). The appeal of prearrest diversion lies in its promise for reducing criminal recidivism in the longer term, enhancing public safety, saving money, and improving access to the appropriate services for people with mental health problems (Heilbrun et al., 2012; Kane, Evans, & Shokraneh, 2018). There has, however, been no systematic evaluation of research to date which has assessed such outcomes, so prearrest diversion is still not deemed to be evidence‐based (Watson, Compton, & Draine, 2017). Nevertheless, various models have been implemented across the Western world. Most popular in the United States and replicated in over 2,700 agencies, *Crisis Intervention Teams* (CIT) are police‐based teams that respond to incidents involving people with possible mental disorder (Taheri, 2014). In other parts of the world, namely, Canada, Australia, and the United Kingdom, coresponse strategies are more dominant (Reuland, Draper, & Norton, 2012), with select approaches being rolled out on a national scale (e.g. Disley et al., 2016). Such interventions involve formal partnerships between police and mental health professionals, who jointly respond to each incident. The Street Triage scheme in England is an example of this approach, with mental health nurses *on call* to provide on‐site or immediate telephone assistance for police officers (Irvine, Allen, & Webber, 2016).

Our research questions focused on people with suspected mental disorder who come into contact with the police:

1. To what extent is their risk of recidivism reduced after diversion into community health services compared with those who are not?
2. To what extent is their mental health improved after diversion into community health services compared with those who are not?
3. What are the economic costs and/or savings associated with prearrest diversion, and to which sectors do they fall?

We particularly wanted to build on previous reviews (Kane et al., 2018; Paton et al., 2016; Shapiro et al., 2015; Scott et al., 2013; Taheri, 2014), which are limited to the impact of prearrest diversion on immediate arrest rates, and so focus on longer term outcomes.

## 2 METHODS

We followed the EPPI Centre (https://eppi.ioe.ac.uk/cms/) stages for systematic reviews. We consulted with stakeholders, including policy makers, police, third sector organisations, academics, and clinical practitioners on the research questions and search terms to ensure that, as far as possible, their needs would be met (Gough, Oliver, & Thomas, 2017).

Inclusion and exclusion criteria are set out in Table 1A. In addition, all included studies had to be in English, from an Organisation for Economic Co‐operation and Development country and published after 1995, the year of an updated circular from the UK Home Office (1995) encouraging interagency working with suspects or offenders with mental disorder. Contemporary mental health services operate within similar frameworks as those developed in the 1990s (see Killaspy, 2006).

**Table 1A:** Inclusion and exclusion criteria for the review

Criteria	Inclusion	Exclusion examples
Population	Adults (aged 18/18+ years) experiencing mental health problems, whether formally diagnosed or not *and* eligible for arrest/detention after coming into contact with police/mental health professionals working with police	Sample of all ages where outcomes are not reported separately by age
Intervention	Pre‐ arrest diversion: On attending an incident, police officers/professionals working with them identify mental disorder and choose to divert rather than arrest, whether to inpatient or community services, the latter including primary health care, social workers or community mental health nurses	Arrests and detentions under section 136 of the Mental Health Act 1983/2007 (England and Wales) Interventions that included both pre and postarrest diversion elements.
Comparison	Treatment as usual (i.e. arrest), postarrest diversion or nondiversion intervention by police.	
Outcomes	Any measure of criminal recidivism, any measure of mental health status or use of mental health services post‐ diversion and/or resource use/costs data	Measures only of referral rates/police decision to arrest at the point of diversion
Study type	Experimental/quasi‐experimental designs *and/or* cost analyses/full economic evaluations	Before and after impact evaluation, without control group or propensity score matching

The literature search was undertaken using 11 electronic databases (Criminal Justice Abstracts, National Criminal Justice Reference Service Abstracts Database, National Police Library, Psychology, PsycArticles, PsycINFO, MEDLINE, Cochrane Central Register of Controlled Trials, ASSIA, EconLit, and Social Science Citation Index), from inception until December 2016, updated April 2016). In addition, three journals (*Mental Health and Criminal Justice*, *Policing: A journal of Policy and Practice*, and *Police Practice and Research: An International Journal*) and 19 policy/practice orientated websites (see Table 1c) were hand searched for the same period. Finally, the reference lists for each included paper were screened for possible inclusion.

Following best practice (Brunton, Stansfield, Caird, & Thomas, 2017), search terms for police and for mental health crisis were identified and strings developed with reference to searches undertaken in previous reviews. The search strings incorporated both free text and controlled/index terms. A sample algorithm is given in Table 1B.

**Table 1B:** Sample search algorithm (for *Criminal Justice Abstracts*)

ti (Police OR policing OR "law enforcement" OR officer* OR YOT OR YOTS OR constable*) OR ab (Police OR policing OR "law enforcement" OR officer* OR YOT OR YOTS OR constable*) OR SU.EXACT("Police") OR SU.EXACT("Law enforcement") OR SU.EXACT("Community policing")
AND
ti (crisis OR crises OR mentally OR Mental* OR psychiatr* OR vulnerab* OR homeless* OR suicid* OR mind) OR SU.exact("mental health care") OR SU.exact("mental disorders") OR SU.EXACT("Suicides & suicide attempts") OR OR SU.EXACT("Behavior disorders") OR SU.EXACT("Psychiatry") OR SU.EXACT("Personality disorders") OR SU.EXACT ("Crisis intervention")

**Table 1C:** Websites selected for hand searching

The Australian Institute of Police Management http://www.aipm.gov.au/
The Barrow Cadbury Trust http://www.barrowcadbury.org.uk/
The Centre for Problem Oriented Policing http://www.popcenter.org/
The Center for Evidence Based Crime Policy http://cebcp.org/
The Department of Health https://www.gov.uk/government/organisations/department-ofhealth
Her Majesty's Inspectorate of Constabulary (HMIC) http://www.hmic.gov.uk/
Home Office https://www.gov.uk/government/organisations/home-office
MIND http://www.mind.org.uk
The National Alliance on Mental Illness http://www.nami.org/
Ministry of Justice https://www.justice.gov.uk/
National Police Chiefs' Council http://www.npcc.police.uk/
National Offender Management Service http://www.justice.gov.uk/about/noms
NICE National Institute for Health and Care Excellence http://www.nice.org.uk/
National Institute of Justice, http://www.nij.gov/Pages/welcome.aspx
The Police Executive Forum http://www.policeforum.org/
Rethink mental Illness http://www.rethink.org/
Revolving Doors Agency http://www.revolving-doors.org.uk/about-us/
The United States Department of Justice http://www.justice.gov/cjs/
Young Minds http://www.youngminds.org.uk/

An adapted version of an existing tool (EPPI Centre, 2007) was used for the data extraction and quality appraisal., modeled on the EMMIE framework (Effectiveness of the intervention; the Mechanism and mediators theorised to be at work; the Moderators that are likely to affect the response to the intervention; Implementation issues in practice and any Economic costs reported. See Johnson, Tilley, & Bowers, 2015).

Effect sizes were calculated for each study where sufficient data allowed. Risk ratios (RR) were calculated for alloutcomes as a meaningful and easily understood metric (Grant, 2014) that iscomparable across studies. All included *effect studies* were critically appraised using an adapted version of a quality assessment checklist for quantitative intervention studies (NICE, 2012; Waddington & Hombrados, 2012; Baird, Ferreira, Özler, & Woolcock, 2013). The synthesis of economic data drew on methods for an economic commentary (Shemilt et al., 2011; Shemilt et al., 2013).

## 3 RESULTS

**Figure 1:**
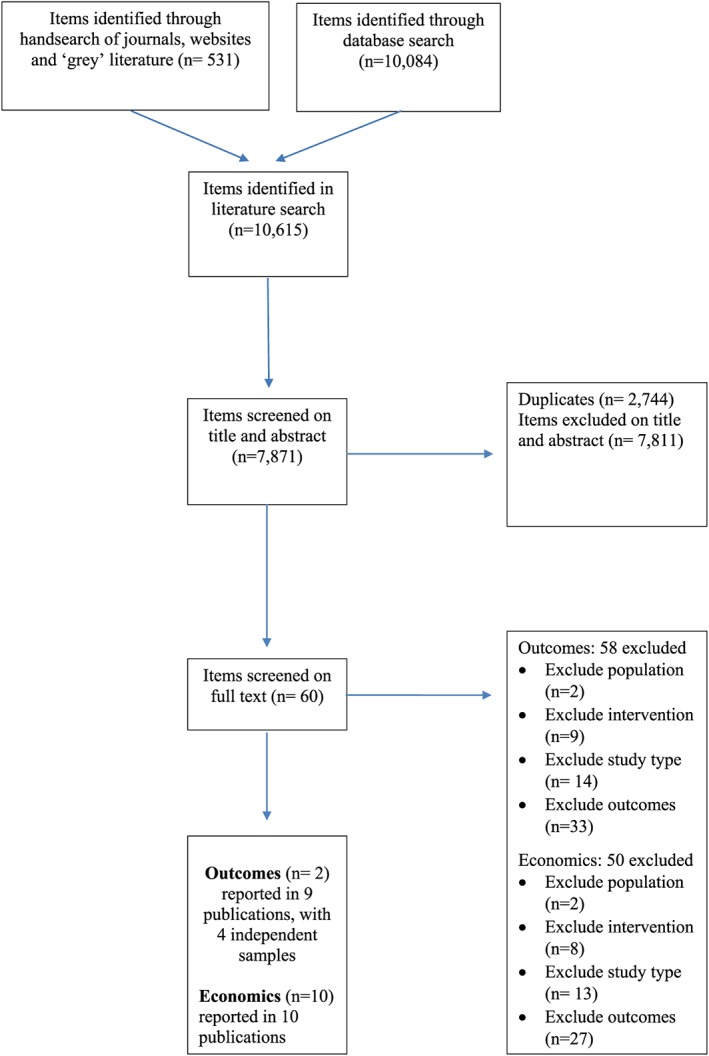
Flow of studies through the review [Colour figure can be viewed at http://wileyonlinelibrary.com]

The literature search identified 7,871 unique titles from all source searches. Figure 1 shows the screening and selection process. Included studies are considered in two main groups: *outcomes* assessing crime and mental health impacts of prearrest diversion and economics containing cost‐data.

### 3.1 Crime and mental health outcomes

Only two studies could be included for the outcomes analysis (Bonkiewicz, Green, Moyer, & Wright, 2014; Broner, Lattimore, Cowell, & Schlenger, 2004), although these, between them, reported on four separate samples in nine publications (see Table 2).

**Table 2:** Longer term mental health or criminological outcomes after pre‐arrest diversion

Study features	Bonkiewicz et al. (2014) link scheme	Broner et al. (2004) CIT, Memphis	Broner et al. (2004) CIT, Portland	Broner et al. (2004), Mobile Crisis Outreach, Philadelphia
Reports informing data extraction	Bonkiewicz et al. (2014)	Broner et al. (2004); Cowell et al. (2004); Lattimore, Schlenger, Strom, Cowell, and Wen Ng (2002); Lattimore et al. (2003); and Steadman et al. (2001)	Broner et al. (2004); Lattimore et al. (2002, 2003); Gratton et al. (2001); and Steadman et al. (2001)	Broner et al. (2004); Lattimore et al. (2002, 2003); and Steadman et al. (2001)
Study design	Matched comparison group, post‐hoc analysis to control for differences	Matched comparison group, post‐hoc analysis to control for differences	Matched comparison group, post‐hoc analysis to control for differences	Matched comparison group, post‐hoc analysis to control for differences
Intervention group (*n*)	166	301	73	64
Control group (*n*)	573	308	132	69
Total (*n*)	739	609	205	133
Data collection	One‐to‐one interviews	One‐to‐one interviews	One‐to‐one interviews	One‐to‐one interviews
Data analysis	Propensity Score Matching	Multivariate analysis	Multivariate analysis	Multivariate analysis
Timing of outcomes after diversion	6 months	3 months 12 months	3 months 12 months	3 months 12 months
Criminal Justice outcomes	Any arrest (Official records)	Any arrest N. of arrests (Self‐report)	Any arrest N. of arrests (Self‐report)	Any arrest N. of arrests (Self‐report)
Effect size (risk ratio) for any arrest	0.68 (95% CI 0.08–5.82)	3 months: 0.983 (95% CI 0.50–1.92) 12 months: 1.369 (95% CI 0.54–3.48)	3 months: 2.252 (95% CI 0.81–6.27) 12 months: 2.982 (95% CI 1.00–8.89)^a^	3 months: 4.32 (95% CI 0.80–23.45) 12 months: 2.046 (95% CI 0.14–29.71)
Mental health outcomes	Number of mental health calls for service Any emergency protective custody incident (Official records)	Mental health status (CSI and SF‐12) Mental health counselling Psychotropic medications Hospitalisation (Self‐report)	Mental health status (CSI and SF‐12) Mental health counselling Psychotropic medications Hospitalisation (Self‐report)	Mental health status (CSI and SF‐12) Mental health counselling Psychotropic medications Hospitalisation (Self‐report)
Effect size (risk ratio) for counselling	‐	3 months: 1.60 (95% CI 0.12–2.50)^a^ 12 months: 1.26 (95% CI 0.80–1.99)	3 months: 1.25 (95% CI 0.60–2.62) 12 months: 1.49 (95% CI 0.76–2.91)	3 months: 0.23 (95% CI 0.11–0.48)^a^ 12 months: 0.77(95% CI 0.32–1.85)
Effect size (risk ratio) for prescription of mental health medications	‐	3 months: 1.23 (95% CI 1.05–1.44)^a^ 12 months: 1.09 (95% CI 0.95–1.26)	3 months: 1.24 (95% CI 1.03–1.48)^a^ 12 months: 1.18 (95% CI 0.96–1.46)	3 months: 1.32 (95% CI 1.07–1.63)^a^ 12 months: 1.34 (95% CI 1.07–1.67)^a^
Effect size (risk ratio) for hospitalisation for mental health condition	‐	3 months: 1.92 (95% CI 1.17–3.16)^a^ 12 months: 1.22 (95% CI 0.65–2.30)	3 months: 2.77 (95% CI 1.28–5.99)^a^ 12 months: 2.03 (95% CI 0.76–5.47)	3 months: 4.84 (95% CI 1.46–16.04)^a^ 12 months: 4.14 (95% CI 1.40–12.18)^a^

*Note*. CI: Confidence Interval; CIT: Crisis Intervention Teams; CSI: Colorado Symptoms Inventory; SF12: Mental health component of short form 12 (SF‐12) Health Survey.

a Statistically significant.

These studies evaluated three different types of intervention: CIT, Link Scheme, and Mobile Crisis Outreach. In these studies, *CIT* as implemented in two different sites included 40 hours of intensive training for police officers and a Crisis Triage Centre (CTC). On responding to incidents, CIT officers sent/took the person with suspected mental disorder to the CTC rather than arresting him/her. The person was then assessed and linked to community mental health/substance abuse services. The Portland CIT also included a case manager and a *boundary spanner* to facilitate multisystem cooperation. The *Link Scheme*, formally named *Post‐Crisis Assistance Programme*, encouraged police officers to identify individuals in a mental health crisis or with undiagnosed problems and link them to appropriate services. Most officers (65% intervention: 80% control group) had undertaken CIT training. Following referral, the person with suspected mental disorder was contacted by a *peer‐specialist* within 24–48 hours, for support to develop a long‐term mental health plan. The *Mobile Crisis Outreach* included several elements: a CTC, *boundary spanners*, and case management. Police attending an incident transported the individual to the CTC or requested ambulance/crisis outreach team attendance.

The models thus shared some features but also had distinct characteristics: both CIT and Mobile Crisis Outreach included referral to CTCs and used dedicated assessment of the person with suspected mental disorder by health professionals. Police training was common to CIT and the Link Scheme.

Table 2 confirms the similarity of design between these studies. Both compared individuals who were diverted with those that were not but rather arrested and/or incarcerated within the study period. Quality rating for the studies was low, with the main weaknesses in selection and performance bias. Participants in Broner et al. (2004) had been diagnosed with severe mental illness and a substance use disorder. In Bonkiewicz et al. (2014: 773), participants did not necessarily have a diagnosed mental illness but had experienced a *mental health crisis* prior to police attendance; most of them were “either exhibiting symptoms of substance dependency or reported a history of substance abuse.”

#### 3.1.1 Crime outcomes

In the short term (3 months after diversion), in one of the three sites of the Broner et al. (2004) evaluation, the intervention group had a reduced risk of arrest compared with controls, but in the other two there was an apparently increased risk of arrest following prearrest diversion. Twelve months after diversion, diverted individuals appeared to have an increased risk of arrest compared with controls at all three sites. The effect sizes were significant only at the Portland site (CIT, 12 months after diversion). The other study (Bonkiewicz et al., 2014) found no significant effect of diversion on arrests up to 6 months after the index police contact.

#### 3.1.2 Mental health outcomes

In the absence of data on mental health status, service use was taken as a proxy measure of mental health. One included study reported outcomes for counselling, medication, and hospitalisation (Broner et al., 2004) with, on the whole, slightly but significantly higher service use for the intervention group.

With respect to specific treatments, there was an increased likelihood that the diverted participants received counselling (three or more sessions) compared with controls for CIT sites; for the Mobile Crisis Outreach team, there was a suggestion that intervention group participants were less likely than controls to receive counselling, but only two of the effect sizes reached statistical significance (CIT, Memphis, and Mobile Crisis Outreach, Philadelphia, at 3 months). For all three sites, effect sizes suggest that prearrest diversion increased the probability that intervention group participants were prescribed psychotropic medications compared with the control group at 3 and 12 months after diversion. In the CIT sites, findings were statistically significant at the 3 month but not 12‐month, follow up. Effect sizes for likelihood of medication in the intervention group were statistically significant, and remained so, in the Crisis Outreach model.

Intervention group participants were more likely to have been hospitalised for a mental health condition, compared with the control group, at 3 and 12 months after diversion. The relative risk of the intervention group being hospitalised for a mental disorder reduced over time. Although all findings were statistically significant at 3 months, the findings of only one site (Mobile Crisis Outreach, Philadephia) reached statistical significance at the 12‐month follow‐up.

### 3.2 Economics studies

Ten eligible papers reported, between them, five independent economic evaluations. Five further studies, of varying design, reported relevant cost‐related information. The five economic evaluations analysed the costs of two distinct population groups, comparing cost of the prearrest diversion with treatment as usual (Cowell, Broner, & Dupont, 2004; Cowell, Hinde, Broner, & Aldridge, 2013; Scott, 2000; Allen Consulting, 2012) or postarrest diversion (Cowell, Hinde Jesse, Broner, & Aldridge, 2015). Only one of these assessed cost‐effectiveness (Cowell et al., 2004) with the remaining four reporting costs only. We did not formally assess the quality of these included studies, but the authors themselves identified concerns about selection bias and the possible nonequivalence of groups (see Table 3).

**Table 3:** Summary of methodological features and findings of included studies for making economic evaluations or cost assessments

Study features	Cowell et al. (2004)	Cowell et al. (2013)	Scott (2000)	Allen Consulting Group (2012)	Cowell et al. (2015)	El‐Mallakh et al. (2014)	Massachusetts Department (2009)	Orr and Pinals (2014)	Parsonage (2009)	Tartaro (2015)
Study design	Economic evaluation: cost‐effectiveness	Economic evaluation: costs only	Economic evaluation: costs only	Economic evaluation: costs only	Economic evaluation: cost only	Cost description	Cost description	Cost description	Economic commentary	Retrospective cohort study
Country	United States	United States	United States	Australia	United States	United States	United States	United States	United Kingdom	United States
Intervention	CIT	CIT	Mobile Crisis Outreach	Mobile Crisis Outreach	CIT	CIT	Multiple: pre‐arrest	Multiple pre‐arrest: CIT, co‐response, innovative models	Multiple Pre and post diversion interventions	pre‐arrest, post‐arrest and re‐entry (on release from prison)
Comparison	Treatment as usual	Treatment as usual	Treatment as usual	Treatment as usual	Post‐arrest diversion	N/A	N/A	N/A	N/A	N/A
Cost categories: crime	The courts, public defenders' and prosecutors' offices, police, prisons (direct criminal justice costs)	Police, courts, prisons (direct criminal justice costs)	Police time spent on programme delivery (direct criminal justice costs)	Police time and equipment (direct criminal justice costs)	Police, courts, and prisons (direct criminal justice costs)	Not reported	Not reported	Not reported	Police, courts, and prisons (direct criminal justice costs)	Police, courts, and prisons (direct criminal justice costs)
Cost categories: health	Inpatient, residential, outpatient treatment for mental health/substance abuse received by clients in the community or in prison (direct health care costs)	Treatment costs of local behavioural health care provider, local hospital system, and medication providers (direct health care costs)	Mental health professional time spent on programme delivery, use of psychiatric hospital in‐patient services (direct health care costs).	Mental health clinician time, ambulance and/or other transport, hospital emergency department use (direct health care costs).	Treatment costs incurred by the local behavioural health care provider, local hospital system, medication providers (direct health care costs)	Not reported	None	Not reported	None	None
Main findings	Diversion associated with higher average (by £4,147) total direct costs per person at 3 months compared with treatment as usual (TAU). Incremental cost‐effectiveness ratio (ICER) of £1,194 per one point improvement on the CSI (95% CI: £475–17,132)	Diversion associated with lower (by 2,240) average total costs per person at 2 years compared with TAU (SE = 655)	Diversion associated with lower (by £445) average total costs per person than TAU	Diversion associated with lower average total cost per case than in all four comparison groups (range £36‐ £203)	Pre‐arrest diversion implementation higher average cost ($370/ £255 per person) than postarrest bond ($238/ £165) or docket ($205/ £142) diversion	Pre‐arrest diversion produced overall cost‐savings from a multisector perspective (criminal justice and health care combined) of $1,024,897/ £707,660,	Total estimated savings from diverting 100 individuals = $ 652, 000/ £ 448, 761	Estimated cost‐savings at state level (>£1.5 M per annum 2011–2014 for effective diversion from prisons and emergency health services	“… it is estimated that [diverting offenders with mental health problems towards effective community‐based services] will save >£23,000 crime‐related costs per case	Estimated an average (mean) cost‐saving accruing to the criminal justice system of £12,562 per diverted client

Three of the five included economic studies found that prearrest diversion led to *cost shifting* from local criminal justice agencies to local health care agencies for up to 2 years but did create overall savings (Cowell et al., 2013; Scott, 2000; Allen Consulting, 2012). This finding was likely driven by a lower proportion of referrals to hospital among diverted clients. All costs are expressed in a common currency and price year: 2016 £‐sterling.

A cost analysis of CIT (USA) found that diversion was associated with lower average total costs per diverted individual at 2 years compared with treatment as usual (£2,240 lower, SE = 655). Higher mental health care treatment costs at 2 years (£499 higher, SE = 545) among diverted individuals, combined with lower costs to the criminal justice system (£2,740 lower, SE = 332), drove the total (Cowell et al., 2013). Cost analysis of a Mobile Outreach Team found that diversion was associated with lower average total costs per individual (£445 lower) compared with treatment as usual. Higher incremental direct costs of implementation (£393 higher) were offset by lower direct health care costs (£847 lower), reflecting the higher probability that people seen by the mobile crisis team were managed without psychiatric hospitalisation (Scott, 2000). Cost analysis of a mobile corresponding model, in Australia, found that the average total cost per case was lower among diverted than nondiverted clients in all four scenarios examined (ranging from £36 lower for the most and £203 lower for least conservative), driven primarily by a fewer referrals to hospital emergency departments, and shorter average length of stay following admission of those diverted (Allen Consulting, 2012).

Five other included studies, with cost‐related description, supported the finding that prearrest diversion can produce overall cost‐savings from a multisector perspective (El‐Mallakh, Kiran, & El‐Mallakh, 2014; Massachusetts Department of Mental Health Forensic Mental Health Services, 2009; Orr & Pinals, 2014; Parsonage, 2009; Tartaro, 2015.

Two of the economic evaluations associated prearrest diversion with higher direct costs. A cost‐effectiveness analysis of a CIT intervention found that police‐based diversion strategies were associated with higher total direct mean costs of £4,147 per diverted individual at 3 months compared with *treatment as usual* (Cowell et al., 2004). In‐patient mental health care costs constituted the main extra expenditure among diverted individuals, which drove this overall finding. An incremental cost‐effectiveness ratio of £1,194 per one point improvement on the CSI (95% CI: 475 to 17,132) was also estimated. One further included study found that prearrest diversion strategies had higher implementation costs than postarrest diversion, which found that the local health care provider incurred 90% of total prearrest diversion implementation costs and local courts 55% and 58% of the costs respectively with postarrest diversion (Cowell et al., 2015).

## 4 DISCUSSION

This is the first systematic review, to our knowledge, to examine the impacts of prearrest diversion on crime, mental health, and costs after the intervention. The economic analysis highlights the promise of prearrest diversion as a strategy that may lead to overall cost savings per diverted individual, compared with treatment as usual, although, as things stand, health services would seem to benefit least from such savings. This is particularly important in the context of austerity measures when system‐wide resources need to be maximised (Parker et al., 2018) and presents an initial step in examining cost‐effectiveness (Kane et al., 2018). In contrast, there is equivocal evidence on the extent to which prearrest diversion improves subsequent mental health outcomes or reduces the risk of reoffending, but only two includable studies across four sites, all in the United States, have been published. It would be important to have access to studies in a much wider range or jurisdictions where diversion is used (Watson et al., 2017). As reviews on early phases of diversion have found, these studies show that prearrest diversion has the potential to initiate links with relevant community services for people with suspected mental disorder (Shapiro et al., 2015; Taheri, 2014) but contribute evidence that such interventions may not necessarily maintain them. There is a suggestion that the severity of mental health problems was greater among diverted individuals that control group participants (Broner et al., 2004; Gratton et al., 2001), which may have constituted a barrier to sustained treatment over the longer term, so “increased oversight and more directive models of diversion” may help for such people (Lattimore, Broner, Sherman, Frisman, & Shafer, 2003: 30). Other aspects that may influence outcomes include the availability of mental health services, timely linkage to treatment, and accurate mental health assessments (Bonkiewicz et al., 2014; Schwarzfeld, Reuland, & Plotkin, 2008; Watson, Ottati, Draine, & Morabito, 2011). The failure of diversion to result in sustained treatment for mental disorder, whatever the reason, may serve to explain the potential for adverse outcomes indicated by this review, for example, a finding that prearrest diversion was associated with an increased longer‐term risk of arrest (CIT, Portland; Broner et al., 2004). With a growing impetus for evaluations to identify potentially harmful effects (Bonell, Jamal, Melendez‐Torres, & Cummins, 2015), future studies need to plan to examine the possibility of unintended outcomes.

The limitations of the evidence base mean that caution is advised when interpreting the findings.

Firstly, the review only identified two *outcomes* studies, evaluating prearrest diversion in four independent sites. With the routine implementation of prearrest diversion in many police districts in many countries, this is surprisingly few (Parker et al., 2018). Although the search generated 60 potentially eligible studies, almost half were excluded on population, intervention, or study type (27). The majority of studies (33) using high‐quality designs had not quantified either criminal or mental health outcomes. Evaluations of more recent interventions, such as street triage, were excluded on this basis. Back in 2009, reviews highlighted the dearth of studies that measured longer term, subject‐level outcomes (Parsonage, 2009; Sirotich, 2009) so it is discouraging still to find limited high‐quality evaluations. Further, our review only identified 10 studies with cost data. Further economic evaluations are needed, which adopt a multisector perspective and capture data over at least 2 years to measure medium‐ to long‐term cost savings (Cowell et al., 2013).

Secondly, real‐world settings, which are notoriously *very difficult to study* (Compton, Bahora, Watson, & Oliva, 2008: 53), and associated methodological challenges in this kind of research may also contribute to the little research and apparent difficulty in finding intervention effects. A lack of group equivalence at baseline, for example, may mean that any effects may have been masked by selection bias. The included studies compared groups that were selected through the criminal justice process and, as other reviews have highlighted, this is inherently problematic (Sirotich, 2009). The size of the samples may inhibit the power to detect effects (Broner et al., 2004; Sirotich, 2009), but further studies are required to explore this possibility.

## 5 CONCLUSIONS

This review is timely given the growing popularity of prearrest diversion amongst policy makers, with a range of models being widely implemented around the world. Finding that there is such a small body of evidence and that there is at least a possibility of adverse outcomes, this review highlights the importance of setting up and FUNDING research into diversion in its various forms alongside any new initiatives or plans to spread existing programmes more widely. Current knowledge is insufficient to inform decision makers or practitioners about the most appropriate methods for maximising the chance of positive outcomes and effective use of resources.

## FUNDING

This work was supported by the UK College of Policing and the Economic and Social Research Council (ESRC). ESRC grant title, ‘University Consortium for Evidence‐Based Crime Reduction’, grant ref: ES/L007223/1.

## CONFLICT OF INTEREST STATEMENT

The authors have no conflict of interest to declare**.**
